# Integrated soil–microbe–plant regulation for maize disease control in the black soil of Northeast China

**DOI:** 10.1002/mlf2.70113

**Published:** 2026-10-28

**Authors:** Pingzhi Zhao, Jiangchun Hu, Jia Zhou, Ru‐Bin Chen, Jian‐Hua Zhao, Chaoying Deng, Yantao Jia

**Affiliations:** ^1^ State Key Laboratory of Microbial Diversity and Innovative Utilization, Department of Agri‐microbiomics and Biotechnology Institute of Microbiology, Chinese Academy of Sciences Beijing China; ^2^ Institute of Applied Ecology, Chinese Academy of Sciences Shenyang China; ^3^ CAS Center for Excellence in Biotic Interactions University of Chinese Academy of Sciences Beijing China

**Keywords:** black soil region, maize disease, microbial regulation, plant immunity, soil–microbe–plant regulation

## Abstract

The black soil region of Northeast China is a critical area for maize production in China. However, the long‐term continuous cropping, coupled with residue‐driven pathogen carryover, soil organic matter depletion, nutrient imbalance, and climate variability, has increased maize vulnerability to diseases. This review synthesizes current knowledge on the integrated regulation of soil, microbiomes, and plant immunity for maize disease management in this region. It assesses the physicochemical properties of the soil, the microbiota, and plant immune responses. Particular attention is paid to current biological control approaches. These plant immunity‐ and microbiome‐based strategies include beneficial microbe‐mediated immune priming, activation of induced systemic resistance, modulation of plant hormone signaling networks, RNA interference (RNAi)‐based immune regulation, and microbiome‐assisted enhancement of disease resistance. The underlying mechanisms include recruitment of beneficial microorganisms, suppression of pathogen growth and virulence, stabilization of protective microbial networks, activation of plant immune signaling, and enhancement of stress resilience. This review also compares these strategies in terms of disease‐control efficacy, yield benefits, stability, cost, biosafety, and commercialization potential, and proposes a region‐specific pathway for their integrated deployment. Establishment of a coordinated regulatory framework that combines soil‐health improvement, microbiome intervention, pathogen monitoring, and plant immune enhancement will support the transition from chemical‐dependent disease control to proactive and sustainable maize production systems in the black soil region of Northeast China.

## INTRODUCTION

The black soil region of Northeast China is a critical grain‐production area and a primary zone for spring maize cultivation. The soils in this region have traditionally facilitated high agricultural productivity due to their substantial humus content, favorable soil aggregation, and significant nutrient‐retention capacity. However, decades of intensive cultivation, frequent tillage, simplified fertilization, soil erosion, and soil organic matter reduction have progressively compromised soil structure and its ecological buffering capacity^1^. In maize, long‐term continuous cropping and residue retention can further promote the carryover of pathogen inoculum, alter nutrient cycling, and disturb soil microbial community structure^2^, ^3^. Together with climate variability, these changes have increased the vulnerability of maize production to multiple diseases in the black soil region.

Maize diseases in Northeast China are no longer determined solely by the presence of individual pathogens but increasingly by the complex interplay among cropping patterns, soil health, residue‐borne inoculum, host susceptibility, and microbiome stability^4^, ^5^. Recent regional surveys indicate that foliar diseases such as gray leaf spot, northern corn leaf blight, *Curvularia* leaf spot, maize eyespot, maize white spot, and stalk rot require continuous monitoring in Northeast China^4^. *Fusarium*‐related disease complexes are particularly important because *Fusarium verticillioides*, *F. graminearum,* and related species can cause stalk rot, root rot, and ear rot, resulting in yield loss, lodging risk, seedling decline, and mycotoxin contamination^6^, ^7^, ^8^, ^9^. These diseases therefore affect not only maize yield but also grain quality and food safety. While conventional control strategies, including the use of resistant cultivars, chemical fungicides, and agronomic management, remain essential, they often prove inadequate when disease pressure is exacerbated by factors such as soil degradation, continuous cropping, and microbiome imbalance^3^, ^10^, ^11^.

Maize disease resistance has traditionally been interpreted through host genetic immunity, including pathogen recognition, defense signaling, antimicrobial metabolites, cell‐wall reinforcement, and resistance responses. However, recent research indicates that the plant‐associated microbiome plays a crucial role as an extension of plant immunity^10^, ^12^. Beneficial microorganisms exert direct inhibitory effects on pathogens via mechanisms such as antibiosis, niche competition, mycoparasitism, siderophore production, and the secretion of lytic enzymes^13^, ^14^, ^15^. Additionally, they indirectly augment host resistance by inducing systemic resistance, priming the immune system, and modulating signaling pathways associated with salicylic acid (SA), jasmonic acid (JA), and ethylene (ET)^13^, ^16^. In maize, root exudates and defense‐related secondary metabolites constitute a crucial interface between host immunity and rhizosphere microbial assembly. For instance, benzoxazinoids such as DIMBOA (2,4‐dihydroxy‐7‐methoxy‐1,4‐benzoxazin‐3‐one) secreted by maize roots can shape bacterial and fungal communities in the rhizosphere, thereby influencing plant–soil feedbacks and enhancing defense‐related responses^17^, ^18^, ^19^. These observations imply that maize resistance is not exclusively governed by the plant genome but also by the host's capability to recruit, sustain, and functionally activate beneficial microbial consortia.

This review highlights recent advancements and strategies for managing maize diseases through integrated soil–microbe–plant systems in the black soil region of Northeast China. It covers the regional disease context and factors influencing disease occurrence, evaluates biological control and plant immune regulation methods, and explores new technologies like synthetic microbial communities (SynComs) and microbiome‐based monitoring. Emphasis is placed on beneficial microbe recruitment, pathogen gene silencing, immune priming, and microbiome‐assisted disease resistance. We also propose a strategic pathway for sustainable maize production in the region.

## OCCURRENCE OF MAIZE DISEASES IN THE BLACK SOIL REGION OF NORTHEAST CHINA

Maize diseases in China constitute a diverse and spatially heterogeneous disease complex. A long‐term analysis of maize diseases and pests across six maize‐growing regions in China from 1999 to 2018 identified 15 major maize disease categories, including maize leaf blight, head smut, rusts, gray leaf spot, *Curvularia* leaf spot, sheath blight, stalk rot, root rot, and downy mildew^5^. It also showed that the overall occurrence of maize diseases and pests increased over the two decades, with the largest average occurrence extents in the northern spring maize region and the Huang–Huai–Hai summer maize region. The Northeast China's black soil region is a particularly important maize‐production area and disease‐risk zone^1^, ^4^. Recent surveys suggest that the disease profile of maize in Northeast China is changing. Field investigations conducted in 2024 and 2025 reported that gray leaf spot and northern corn leaf blight were the most prevalent foliar diseases, with gray leaf spot now appearing as the predominant disease in many surveyed areas^4^. Stalk rot incidence was generally low, although Liaoning Province showed relatively higher levels than Jilin Province and Heilongjiang Province. Other diseases, such as *Curvularia* leaf spot and maize eyespot, were more localized or unstable. Maize white spot was reported as an emerging threat, with severe outbreaks and northward expansion in Liaoning Province and Jilin Province during recent warm and humid summers. Overall, in the Northeast China's black soil region, the disease spectrum appears to be shifting from the historical importance of northern corn leaf blight toward a more complex foliar–disease assemblage dominated by gray leaf spot and newly emerging diseases such as maize white spot, while *Fusarium* ear and stalk rots remain important for yield stability, lodging resistance, and mycotoxin safety^4^, ^6^, ^7^, ^8^.

Disease occurrence in the Northeast China's black soil region is influenced by both regional climate and local production practices^4^. Warmer and wetter conditions in Liaoning Province and Jilin Province compared with those in Heilongjiang Province, together with mountainous terrain that promotes dew formation and localized high humidity, appear to favor gray leaf spot and other foliar diseases. The predominance of rain‐fed maize also means that interannual variations in precipitation can create either favorable canopy humidity for foliar pathogens or waterlogging and plant stress that increase susceptibility to stalk and ear diseases. In Jilin Province, *Fusarium* ear rot surveys showed strong spatial heterogeneity: *F. graminearum* and *F. boothii* were more important in eastern and western areas, whereas *F. verticillioides* was dominant in central areas, indicating that pathogen composition varies across black soil subregions^8^. Agronomic factors, including continuous maize cultivation, residue retention or straw return, cultivar resistance, planting density, seed treatment, and fungicide use, further modulate disease pressure by affecting inoculum survival and host susceptibility^2^, ^3^. Future disease management in this region should therefore integrate regional disease surveillance, pathogen identification, resistant cultivar deployment, residue and crop‐rotation management.

## SOIL–MICROBE–PLANT REGULATION FOR MAIZE DISEASE CONTROL

Soil–microbe–plant interactions are crucial for managing maize disease by connecting soil health, microbial dynamics, and plant defenses^10^, ^11^. In Northeast China's black soil region, intensive farming practices have significantly changed soil properties, microbial communities, and disease pressure^1^, ^3^. Maize disease is influenced by pathogen levels and the stability of the soil–microbiome–plant system. Key processes include pathogen survival in residues, microbial imbalances from continuous cropping, rhizosphere selection by plants, beneficial microbe recruitment, nutrient cycling, and microbial network stability (Figure 1)^3^, ^11^, ^14^. An understanding of these interactions aids in developing integrated disease‐management strategies, shifting focus from targeting single pathogens to fostering a resilient, disease‐suppressive microbiome^11^, ^20^.

**Figure 1:**
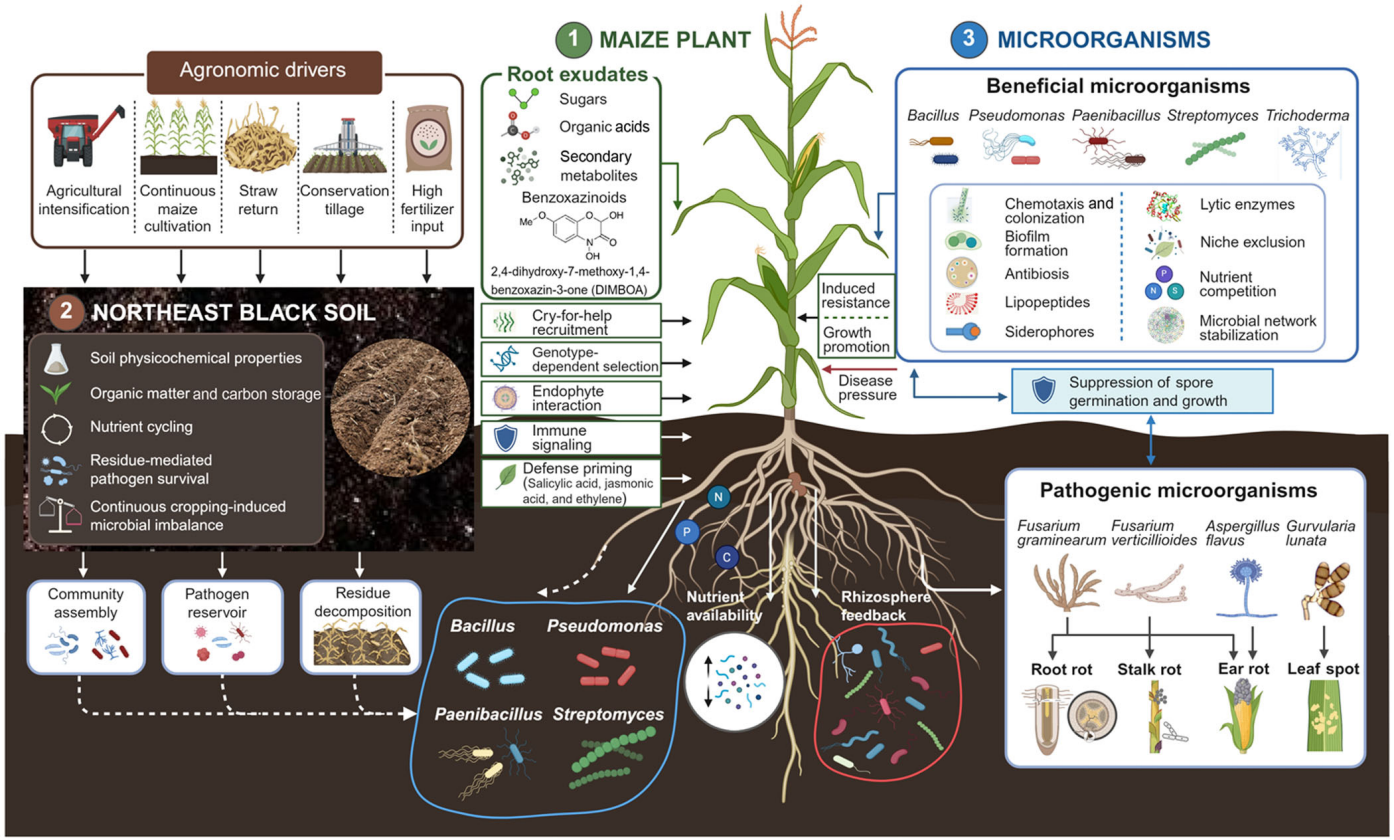
Soil–microbe–plant regulation of maize disease in the black soil region of Northeast China. Long‐term agronomic practices, including agricultural intensification, continuous maize cultivation, straw return, conservation tillage, and high fertilizer input, reshape soil physicochemical properties, nutrient cycling, residue‐mediated pathogen reservoirs, and continuous cropping‐induced microbial imbalance. Maize roots release exudates, including sugars, organic acids, and secondary metabolites such as benzoxazinoids, which mediate genotype‐dependent microbial selection and “cry‐for‐help” recruitment under pathogen stress. Beneficial microorganisms, including *Bacillus*, *Pseudomonas*, *Paenibacillus*, *Streptomyces*, and *Trichoderma*, suppress pathogens through colonization, biofilm formation, antibiosis, lipopeptides, siderophore production, lytic enzymes, niche exclusion, nutrient competition, and microbial network stabilization. These processes reduce the impact of pathogenic fungi such as *Fusarium*, *Gibberella*, and *Curvularia*, thereby contributing to the regulation of maize root, stalk, and ear rot. Created by BioRender. Zhao P. (2026) https://BioRender.com/mkwwmdf.

### Soil–microbe interactions in the regulation of maize disease

Soil–microbe interactions are fundamental to the regulation of maize disease because the rhizosphere represents the primary ecological interface where maize roots, soil physicochemical properties, pathogens, and beneficial microorganisms interact^10^, ^11^. In the black soil region of Northeast China, long‐term agricultural intensification, continuous maize cultivation, straw return, conservation tillage, and increasing fertilizer input have strongly reshaped soil microbial community assembly and disease pressure^1^, ^2^, ^3^. Soil microorganisms, including bacteria, fungi, and archaea, are sensitive indicators of soil condition and are essential for nutrient cycling, organic matter decomposition, soil aggregation, and plant health maintenance^10^, ^21^. Conservation tillage has been widely considered an alternative strategy for mitigating black soil degradation, but its influence on microbial communities and maize disease is complex^3^, ^21^. For example, no tillage with residue return can generate distinct bacterial and archaeal community structures and enrich taxa such as *Bradyrhizobium*, *Candidatus* Solibacter, and *Reyranella*, as well as functional genes associated with ATP‐binding cassette transporters and quorum‐sensing pathways^21^. These microbial changes may enhance nutrient transformation and microbial communication, thereby improving soil ecological function. However, the same practices may also alter residue decomposition dynamics and pathogen survival, indicating that soil health improvement and disease suppression are not always automatically synchronized.

A key function of soil–microbe interactions in maize disease regulation is the control of soil‐ and residue‐borne pathogen reservoirs^3^, ^14^. In Northeast China, long‐term no tillage combined with maize straw retention significantly altered soil bacterial and fungal communities, and increased the abundance of *F. graminearum* and *F. verticillioides*, two important pathogens associated with maize root rot, foot rot, and stalk rot^16^. In a 10‐year field experiment in Lishu County, Jilin Province, no tillage with 0%, 33%, 67%, and 100% of 7.5 t ha^−1^ maize straw mulching increased fungal richness and changed the composition of both fungal and bacterial genera, while the 33% and 100% straw‐mulching treatments produced the strongest increases in *Fusarium* abundance^3^. These results suggest that straw return and conservation tillage can improve soil carbon storage and physical structure, but may also enhance pathogen persistence when residue degradation and microbial antagonism are insufficient^3^, ^20^. Similarly, no tillage with residue mulching in the Sanjiang Plain increased bacterial alpha diversity and improved soil organic carbon and nutrient contents, but its effects on disease‐related microbial groups may depend on residue amount, soil moisture, aeration, and rhizosphere conditions^21^. Therefore, residue management should be optimized not only for soil conservation but also for reducing pathogen inoculum accumulation and promoting antagonistic microbial functions.

Another major function of soil–microbe interactions is the maintenance of microbial diversity, functional redundancy, and disease‐suppressive capacity^11^, ^14^. Continuous maize cultivation can shift the rhizosphere toward a disease‐conducive state by enriching potential pathogens such as *Fusarium*, *Gibberella*, *Curvularia,* and *Cladosporium*, while reducing beneficial or biocontrol‐associated taxa such as *Haliangium* and *Streptomyces* ^2^. In contrast, diverse microbial communities can restrict pathogen establishment through nutrient competition, niche exclusion, antibiosis, parasitism, and activation of plant defense^13^, ^14^. Beneficial genera such as *Bacillu*s, *Pseudomonas*, *Paenibacillus*, and *Streptomyces* are commonly associated with disease‐suppressive soils and can inhibit maize pathogens by producing antifungal metabolites, siderophores, lytic enzymes, and volatile compounds^14^, ^15^, ^22^. Agronomic practices can further reshape disease‐related microbial communities. For example, biochar application and maize–soybean intercropping in black soil improved the rhizosphere environment, enriched beneficial bacteria such as *Arthrobacter*, *Sphingomonas*, and *Nocardioides*, and reduced potentially harmful fungi including *Cladosporium*, *Gibberella*, and *Didymella* ^23^. Therefore, maize disease management in Northeast China's black soil region should integrate optimized straw return, diversified cropping, organic amendments, biocontrol microbes, and pathogen monitoring to reconstruct a resilient and disease‐suppressive rhizosphere microbiome.

### Plant–microbe interactions in the regulation of maize disease

Plant–microbe interactions are central to the regulation of maize disease because maize plants do not function as isolated hosts, but as holobionts associated with rhizosphere microorganisms, root endophytes, phyllosphere microbes, and seed‐borne microbial communities^10^, ^12^. In this regulatory system, microorganisms can directly suppress pathogens through antibiosis, nutrient competition, niche exclusion, mycoparasitism, and production of lytic enzymes or siderophores, while maize plants actively shape microbial community assembly through root exudates, immune signaling, genotype‐dependent selection, and developmental stage‐specific nutrient release^13^, ^14^, ^17^, ^24^. Therefore, maize disease resistance should be understood not only as a consequence of host genetic resistance but also as an ecological outcome of plant‐associated microbiome assembly. In black soil and other intensive maize‐production systems, maize genotype, soil type, environmental conditions, and agricultural practices can strongly influence microbial composition and function, thereby affecting both plant growth and disease pressure^2^, ^3^, ^21^. These interactions provide a biological basis for developing sustainable disease‐management strategies that enhance beneficial microbiota rather than relying only on direct pathogen elimination.

A key mechanism of plant–microbe regulation is the plant‐mediated recruitment of beneficial microorganisms under pathogen stress, often described as a “cry‐for‐help” response^11^, ^25^. Maize roots can modify exudate composition after pathogen infection, thereby attracting protective bacteria with strong chemotaxis, swarming, biofilm formation, and colonization abilities. For example, *Bacillus amyloliquefaciens* OR2‐30 showed stronger chemotaxis toward maize root exudates after *F. graminearum* infection; infected root exudates promoted bacterial swarming, biofilm formation, and rhizosphere colonization, while OR2‐30 suppressed *F. graminearum* by producing iturin‐type lipopeptides that inhibited conidial formation and germination^25^. Similarly, maize‐associated bacteria such as *Bacillus*, *Pseudomonas*, and *Paenibacillus* have been identified as important antagonists against *Fusarium* diseases^15^. In a large‐scale screening study, 11,520 culturable maize rhizospheric bacterial isolates were established, and 42 isolates inhibited *F. verticillioides* growth by more than 45%, with several *Bacillus* strains showing strong in planta activity. These antagonistic effects were associated with glucanases, proteases, chitinases, siderophores, and auxin production. Beneficial fungi can also participate in plant–microbe disease regulation. For instance, *Trichoderma harzianum* T22 colonization of maize roots reduced symptoms caused by *F. verticillioides* and activated SA‐ and JA/ET‐related defense responses^13^, ^16^, indicating that microbial regulation includes both direct pathogen inhibition and priming of host immunity.

Endophytic microorganisms provide another important layer of plant–microbe regulation because they colonize internal maize tissues and interact closely with host metabolism, immunity, and pathogen invasion^10^, ^12^. The maize endophyte *Paenibacillus peoriae* 3‐B4 was reported to control *F. verticillioides*‐induced seedling blight through multiple mechanisms, including inhibition of pathogen infection structures, potential antimicrobial compound production, activation of plant defense responses, and modulation of microbial communities^26^. In pot experiments, the disease index decreased from 80.34% in the pathogen‐only treatment to 20.56% after *P. peoriae* pretreatment, corresponding to a biocontrol efficacy of 74.41%. Recent microbiome and metabolomics studies further indicate that maize resistance to stalk rot is associated with root‐associated microbial networks and rhizosphere metabolites^27^, ^28^. Resistant and susceptible maize lines differ in rhizosphere and root endophytic microbial compositions, and *Fusarium* can become a highly central taxon in diseased root endophyte networks. In resistant lines, enriched metabolites and specific microbial taxa may jointly contribute to disease suppression. Overall, plant–microbe regulation of maize disease is a dynamic and bidirectional process: Beneficial microorganisms suppress pathogens and enhance host defense, while maize plants selectively recruit and maintain protective microbiomes.

## MICROBIAL REGULATION FOR MAIZE DISEASE MANAGEMENT

The overreliance on conventional chemical pesticides has caused a range of adverse outcomes, including environmental contamination, hazardous residues, and the emergence of pathogen resistance. These limitations highlight the need for more sustainable and ecologically based disease‐management strategies^11^, ^20^. Microbial regulation has therefore gained increasing attention as an effective approach for improving soil health, enhancing ecosystem stability, and supporting sustainable crop production^10^, ^11^, ^13^. In soil–microbe–plant systems, microorganisms participate in nutrient cycling, organic matter decomposition, soil aggregation, and carbon turnover, while beneficial microorganisms also promote plant growth and strengthen disease resistance^10^, ^21^. By integrating soil ecological processes with plant health regulation, microbial regulation provides a promising foundation for reducing pesticide dependence and advancing sustainable agriculture and ecological restoration. Therefore, effective maize disease management needs integrated systems that blend multiple technologies with the soil–microbe–plant system (Figure 2).

**Figure 2:**
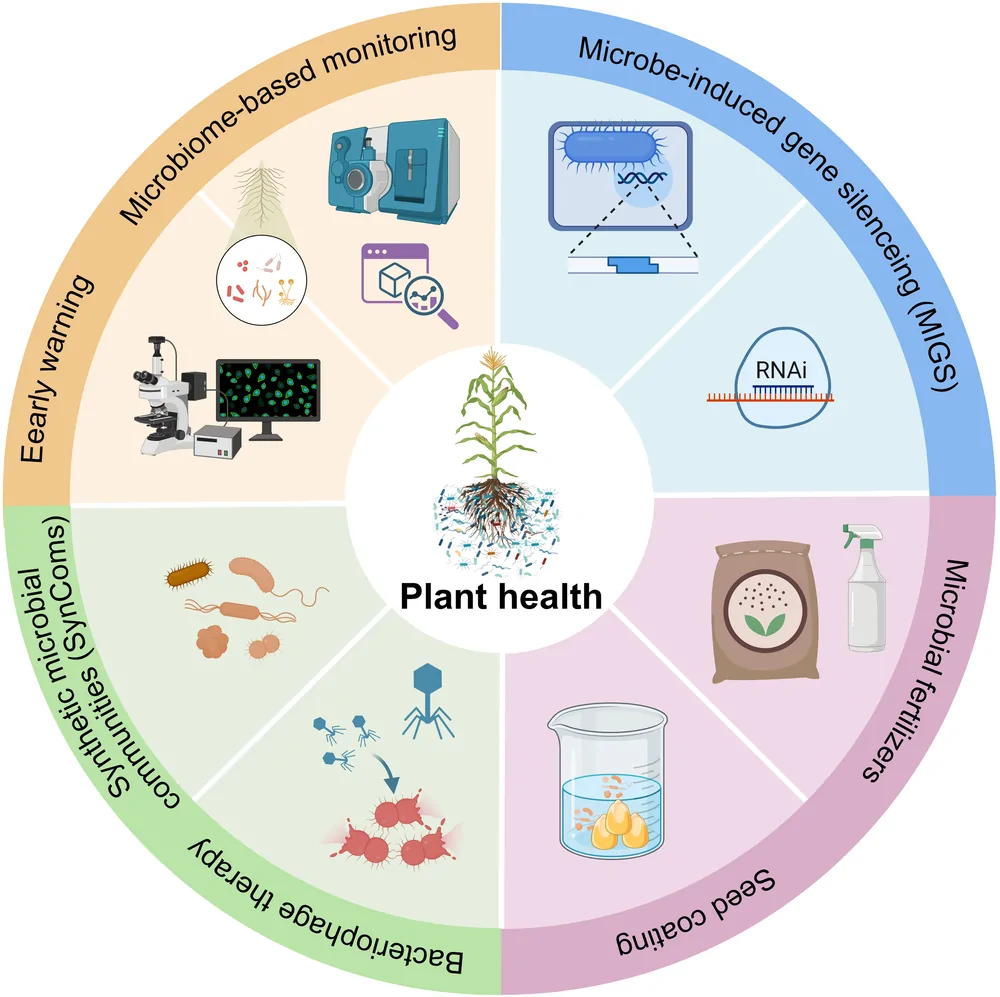
Integrated microbial regulation technologies for maize disease management. The schematic summarizes complementary strategies centered on plant health, including microbiome‐based monitoring, early warning, microbe‐induced gene silencing, synthetic microbial communities, bacteriophage therapy, seed coating, and microbial fertilizers. These approaches target different stages of disease prevention and control by detecting microbiome imbalance, suppressing pathogens, regulating pathogen gene expression, enhancing beneficial microbial consortia, and promoting soil–microbe–plant interactions. Their coordinated deployment supports sustainable maize production with reduced dependence on chemical pesticides. Created by BioRender. Zhao P. (2026) https://BioRender.com.

### Current industrial application of microbial products in maize disease management

The Chinese biocontrol‐agent industry is mainly organized into two product categories: microbial pesticides and microbial fertilizers with biocontrol functions. They are already applied as seed treatments, soil amendments, or foliar sprays, and their commercial forms include wettable powders, granules, liquid suspensions, and seed‐coating formulations^20^, ^29^. The dominant organisms are *Bacillus* spp., *Trichoderma* spp., *Pseudomonas* spp., *Streptomyces lydicus,* and *Pythium oligandrum* ^13^, ^14^, ^15^, ^16^. Driven by supportive policies and market demand for sustainable practices, China's microbial products have expanded rapidly (China Pesticide Information Network https://www.chinapesticide.org.cn). In practical maize production, microbial products are mainly used as seed treatments, soil‐applied microbial fertilizers, straw‐decomposition inoculants, foliar biopesticide sprays, and biological components of “one‐spray multi‐purpose” green‐control programs^29^, ^30^. In the 2025 national maize disease‐control guidance, *B. subtilis* and jinggangmycin were recommended among spray options for foliar diseases at early disease stages. However, the adoption of microbial agents for maize disease management remains limited. As of November 2025, only 3 out of 382 registered fungicides for corn diseases in China are microbial agents, highlighting a significant gap between research potential and commercial deployment (China Pesticide Information Network).

Table 1 lists representative commercial microbial products for maize disease management, which mainly represent three mainstream technological routes: single‐strain *Bacillus* or *Streptomyces* products, filamentous microbial‐antagonist products based on *Trichoderma* or *Pythium oligandrum*, and mixed or symbiotic products combining bacterial and fungal functions^15^, ^16^, ^29^. Their limitations are also clear. Single‐strain products are easy to produce and register, but their field performance may be unstable under heterogeneous black soil conditions^20^, ^31^. Fungal antagonists often colonize roots effectively, but quality control and shelf‐life can be challenging^16^, ^29^, ^32^. Composite formulations may provide broader mechanisms, but they require compatibility testing to avoid antagonism among strains^33^, ^34^, ^35^.

**Table 1:** Representative commercial microbial products for maize disease management.

Microbial species	Formulation	Function	Application method	Registered region	Product No.
*Bacillus subtilis* (strain QST 713)	Aqueous suspension (1 billion CFU/g)	Suppression of northern corn leaf blight	Foliar spray	China	PD20211364
*Bacillus methylotrophicus* (strain 9912)	Wettable powder (3 billion CFU/g)	Disease suppression and growth promotion	Aerial application/spray	China	PD20181602
*Trichoderma harzianum* (strain TH10) with *Bacillus velezensis* (strain T2)	Liquid suspension (strain TH10, 50 million CFU/g; strain T2, 2 billion CFU/g)	Suppression of seed‐ and soil‐borne fungal pathogens	Seed coating	Argentina	SENASA No. 21.617
*Streptomyces lydicus* (strain WYEC108)	Powder (10 million CFU/g)	Suppression of soil‐borne and foliar diseases	Soil/foliar application	International	EPA Reg. No. 73314‐1
*Pythium oligandrum* (strain DV74)	Microbial agent (1 million CFU/g)	Broad‐spectrum pathogen suppression	Seed/soil treatment	USA, China	EPA Reg. No. 81606‐2

For soil‐borne diseases in black soils, commercial use is moving from single microbial products toward residue‐microbe and soil‐health‐oriented applications. A Chinese Mollisol study in Jilin Province showed that combining maize straw with *B. amyloliquefaciens* JF‐1 improved soil aggregation, organic carbon, humic substances, and beneficial microbial taxa such as *Sphingomonas*, *Mortierella*, *Bacillus*, and *Pseudomonas*, while reducing *Fusarium* and *Plectosphaerella,* and alleviating *F. graminearum* damage to maize seedlings^30^. Research‐oriented products also support industrial development, for example, *T. asperellum* GDFS1009 granules controlled maize stalk rot in field trials and increased yield^29^. However, this product's performance is influenced by soil pH, moisture, organic matter availability, background microbiome, pesticide compatibility, storage viability, and application timing^20^, ^29^, ^32^. In the black soil region, low‐temperature sowing, large‐scale mechanized farming, residue return, and spatial heterogeneity of soil fertility all affect inoculant establishment^1^, ^3^, ^21^. Therefore, successful commercialization requires more than selection of an antagonistic strain. It requires formulation, local field validation, diagnostic matching of product to soil and disease risk, and extension services that teach farmers how to integrate microbial products with seed treatment, residue management, and fertilization.

### SynComs for maize disease control

The SynComs are emerging as a next‐generation microbiome‐based strategy for crop disease management because they move beyond single‐strain inoculants toward functionally designed consortia^33^, ^34^. A recent methodological review described SynCom construction as involving core microbiome mining, isolation and culture, functional screening, community assembly, and experimental verification, although standardized construction and field‐validation frameworks are still developing^36^. The mechanisms of SynCom‐mediated disease control include direct antagonism, nutrient and niche competition, induction of systemic resistance, improvement of root nutrient acquisition, and modification of the rhizosphere metabolome. A recent multi‐omics study of corn stalk rot (CSR)‐resistant and ‐susceptible maize cultivars showed that resistant cultivars reshaped their microbiota and selectively recruited *Bacillus* species from the rhizosphere to root and stem compartments, where these bacteria suppressed disease through niche pre‐emption, antimicrobial compound production, and host‐defense activation^28^. This study provides an important mechanistic clue for SynCom design. Three *Bacillus*‐based synthetic communities, SC‐I, SC‐II, and SC‐III, were constructed by combining representative isolates with different anti‐*F. graminearum* phenotypes; greenhouse experiments showed that all three SynComs significantly reduced CSR incidence, while SC‐III, despite lacking direct *in vitro* antagonism, induced maize tyrosine decarboxylase 1 (TYDC1) expression and promoted isoquinoline alkaloid/berberine‐mediated disease suppression. This finding is particularly relevant to Northeast China's black soils because disease management in this region must consider not only pathogen inhibition but also host genotype, residue return, microbial colonization, and soil ecological function.

Although true SynCom field products for maize disease control in Northeast China's black soils have not yet been widely reported, related studies support the feasibility of microbiome engineering. For example, single biocontrol strains such as *B. siamensis* GL‐02 and *T. asperellum* GDFS1009 have shown substantial suppression of maize stalk rot, providing candidate taxa and mechanisms for future cross‐kingdom SynCom design^29^, ^37^. For Northeast China, SynCom design should be linked to soil management. Residue return and reduced tillage may supply carbon substrates and habitat continuity, but they may also increase residue‐borne *Fusarium* inoculum if decomposition is slow. A SynCom intended for such fields should therefore include organisms that accelerate residue decomposition, compete with *Fusarium* during saprophytic growth, and colonize maize roots during early seedling stages. Organic amendments, biochar, or humic substances may serve as carriers and ecological scaffolds for SynCom establishment^20^, ^30^. Key research needs include identifying keystone taxa and functions across black soil subregions, building standardized compatibility tests, developing strain‐tracking tools, and evaluating multi‐season persistence. Because SynComs contain multiple living organisms, regulatory assessment should consider not only individual strain safety but also community‐level behavior, gene exchange potential, and effects on native microbial networks.

### Bacteriophage therapy for maize bacterial diseases

Bacteriophages are viruses that infect bacteria and can lyse specific bacterial pathogens. Compared with broad‐spectrum antibacterial compounds, phages are highly specific and can reduce bacterial populations while preserving much of the non‐target microbiome^38^, ^39^. Phage‐like activity was first observed by Ernest Hankin in 1896, when he reported antibacterial activity against *Vibrio cholerae*. Phages were described independently by Frederick Twort in 1915 and Félix d'Hérelle in 1917, and were used as antibacterial agents called phage therapy in the 1920s^40^. Their importance in agriculture has increased significantly due to the rising challenge of antibiotic‐resistant bacteria and the growing demand for residue‐free food products. In 2005, the first bacteriophage product AgriPhage^TM^ of OmniLytics Inc. received Environmental Protection Agency (EPA) approval in the United States^39^. These naturally occurring bacteriophages were used for the treatment of bacterial spot in tomatoes and speck in peppers. In July 2025, the European Commission announced a Commission Implementing Regulation concerning the authorization of a preparation of the bacteriophages as a feed additive for poultry^41^.

Historically, maize was one of the earliest crop systems in which phage therapy was tested: Phage treatment of *Pantoea stewartii*‐infested corn seed reduced Stewart's wilt incidence from 18% to 1.4% in an early field study^41^. More recent maize‐related evidence comes from Goss's wilt, where CN8 bacteriophages formulated in polyvinyl alcohol/whey‐protein seed coatings remained active during storage and significantly reduced *Clavibacter michiganensis* subsp. nebraskensis loads on contaminated maize seeds and seedlings, without impairing germination^42^. Although these diseases are not the only threats in Northeast China, increasing climate variability and seed movement make them relevant to risk preparedness in maize production^4^, ^43^. Overall, bacteriophage therapy should be viewed as a component of integrated bacterial disease management in Northeast China rather than a stand‐alone solution. Its future value will depend on coupling pathogen surveillance, local phage libraries, seed‐health testing, resistant hybrids, residue management, drainage improvement, and microbiome‐friendly formulations. Although direct field evidence from the black soil region is still lacking, studies on Stewart's wilt, Goss's wilt, Xanthomonas diseases, and Dickeya soft rot provide a strong experimental foundation for developing phage‐based, low‐residue precision antibacterial tools for local maize production.

### Microbe‐induced gene silencing (MIGS) for maize disease management

RNA interference (RNAi), also known as RNA silencing, is a conserved mechanism for gene expression regulation in eukaryotic organisms^44^. This process is initiated by double‐stranded RNA (dsRNA), which is subsequently processed into small RNAs (sRNAs). These sRNAs facilitate the degradation of messenger RNA (mRNA), inhibit translation, or promote DNA methylation of target genes. Recent studies have shown that these sRNAs can be transferred across species to induce gene silencing. For instance, plants can deliver their sRNAs to pathogens, where they suppress virulence‐related gene expression, thereby enhancing disease resistance^45^. Therefore, RNAi‐based crop protection has developed rapidly through host‐induced gene silencing (HIGS), spray‐induced gene silencing (SIGS), and MIGS. MIGS uses a beneficial microbe as a living chassis that produces dsRNA or sRNAs targeting essential or virulence‐related genes of a pathogen^46^. The engineered microbe colonizes the rhizosphere‐ or plant‐associated niche, releases RNA molecules, and triggers silencing in the pathogen after uptake. Compared with transgenic host‐induced approaches, MIGS can avoid maize genome transformation and may provide a renewable local source of RNA molecules.

The relevance of MIGS to black soil maize disease control has increased because engineered *T. harzianum* strains have been shown to protect crops against soil‐borne fungal pathogens, and a recent maize study used an RNAi‐engineered *T. harzianum* strain targeting *F. graminearum* to reduce disease severity in maize seedlings^46^, ^47^. Mechanistically, the chassis microbe can contribute conventional biocontrol functions, while the RNAi cargo adds pathogen gene specificity. Candidate target genes should be conserved, essential for pathogenicity, exhibit low off‐target similarity to maize and beneficial microorganisms, and be prioritized in multi‐target designs to raise the evolutionary barrier against rapid escape mutations. Despite its potential, MIGS faces substantial challenges^48^, ^49^. A comprehensive assessment of the ecological implications of introducing engineered microbes into the environment is imperative, particularly concerning potential risks such as horizontal gene transfer and the disruption of indigenous microbial communities. Enhancing the efficiency of dsRNA expression, secretion, and uptake is vital for achieving high efficacy. Furthermore, the development of cost‐effective, scalable production methods and the establishment of clear, scientifically grounded regulatory frameworks for these living RNAi products are critical obstacles that must be addressed to facilitate their commercialization.

Overall, microbial fertilizers and single‐strain biocontrol agents have the highest commercialization maturity and the lowest regulatory barriers, but their disease‐control stability is often moderate (Table 2). SynComs offer broader mechanisms and may be more robust in heterogeneous fields, but their production, quality control, and registration are more complex. Phage therapy is precise and environmentally attractive for bacterial diseases, but it requires pathogen diagnosis and careful formulation. MIGS is mechanistically powerful and highly specific for fungal pathogens, but it is still at an early regulatory and field‐validation stage.

**Table 2:** Comparative assessment of major microbial regulation technologies for maize disease management in Northeast China's black soil systems.

Technology	Main target in maize	Dominant mechanism	Evidence/maturity	Expected field benefit	Cost and stability profile	Major risks	Potential applications
Microbial fertilizer	*Fusarium* stalk/root rot, seedling diseases, and general plant vigor	Antibiosis, competition, nutrient mobilization, and ISR	Commercially mature; many field and product examples	Moderate disease reduction; yield benefit depending on the soil and season	Low to medium cost; moderate stability with good formulation	Variable efficacy, viability loss, and farmer misuse	Baseline preventive seed/soil treatment, especially with soil‐health improvement
SynComs	Complex soil‐borne disease complexes and continuous‐cropping stress	Complementary functional guilds, niche occupation, metabolic cross‐feeding, and ISR	Rapidly developing; fewer standardized commercial products	Potentially higher robustness than single strains; yield stability more important than one‐season yield spike	Medium to high cost; formulation and QC: challenging	Community instability, registration complexity, and interaction uncertainty	High‐risk fields with long‐term monoculture and low biological buffering capacity
Bacteriophage therapy	Bacterial diseases such as Goss's wilt or bacterial leaf streak	Specific bacterial lysis; reduced by cocktails resistance	Proof‐of‐concept and seed‐coating studies; more crop‐specific validation needed	High specificity when pathogen diagnosis is accurate	Cost medium; environmental stability requiring encapsulation or protective carriers	UV/desiccation sensitivity, narrow host range, and resistance	Targeted seed or foliar treatment after bacterial‐risk diagnosis
MIGS	*Fusarium* graminearum and other fungal pathogens	dsRNA/sRNA produced by engineered beneficial microbe to silence pathogen virulence genes	Frontier technology; maize evidence emerging	Potentially strong and specific disease suppression	High development and regulatory cost; formulation: still immature	GMO biosafety, RNA delivery efficiency, and regulatory uncertainty	Stepwise field trials for severe stalk‐rot zones after biosafety assessment

dsRNA, double‐stranded RNA; GMO, genetically modified organism; ISR, induced systemic resistance; MIGS, microbe‐induced gene silencing; QC, quality control; sRNA, small RNA; SynComs, synthetic microbial communities; UV, ultraviolet.

### Microbiome‐based monitoring and early warning for disease outbreaks

Recent advances suggest that microbiome‐based disease prediction is rapidly progressing from conceptual frameworks toward operational agricultural applications. Microbial community shifts can precede visible disease symptoms. Disease‐conducive soils often show simplified microbial networks, reduced beneficial taxa, or increasing abundance of pathogen‐associated fungi and bacteria. Several studies have demonstrated that machine learning models integrating microbiome profiles can accurately predict disease occurrence across diverse agricultural systems. For example, in metadata‐based analyses of disease‐suppressive soils, machine‐learning models distinguished disease‐suppressive from disease‐conducive soils using 54 bacterial biomarker genera, among which 28 genera were considered potentially beneficial^50^. These suppressive soils were generally enriched in *Firmicutes* and *Actinobacteria*, but depleted in *Proteobacteria* and *Bacteroidetes*; functional validation further confirmed the disease‐suppressive potential of *Kribbella*, *Nocardioides*, and *Bacillus*, partly through activation of pathogen‐associated molecular pattern‐triggered immunity. Cross‐system prediction has also been demonstrated for *Fusarium* wilt. A large‐scale meta‐analysis integrated 24 independent bacterial datasets containing 758 samples and 22 independent fungal datasets containing 279 samples from healthy and *Fusarium* wilt‐diseased soils^51^. These samples covered six crops and eight countries, and the random forest model selected 45 bacterial operational taxonomic units (OTUs) and 40 fungal OTUs as predictive indicators, classifying soil health status with >80% accuracy. Diseased soils were associated with higher abundances of *Xanthomonadaceae*, *Bacillaceae*, *Gibberella*, and *F. oxysporum*, whereas healthy soils were enriched in *Streptomyces*, *Bradyrhizobiaceae*, *Comamonadaceae*, *Mortierella*, and nonpathogenic *Fusarium* taxa. More recent deep‐learning meta‐analysis further improved the prediction of soil‐borne fungal diseases. By integrating denoising and data‐balancing strategies, bacterial amplicon sequence variant (ASV)‐based models outperformed fungal ASV‐based models and achieved over 90% accuracy for predicting *Fusarium* and *Verticillium* wilt on independent test datasets^31^. The optimized framework reported 94.46% accuracy in the three‐class model, while bacterial biomarkers such as *Chitinophagaceae*, *Nocardioides*, and *Sphingomonas* were identified as useful indicators for distinguishing healthy and diseased soils. Together, these findings show that microbiome‐based disease prediction does not depend on a single universal microbial marker. Instead, prediction accuracy is improved when models integrate multiple taxonomic indicators, pathogen‐associated taxa, beneficial microbial groups, and community‐level patterns.

Microbiome‐based early warning for maize diseases is still at an exploratory stage, but existing studies show that maize‐associated microbial communities can provide quantitative signals of disease risk across residues, kernels, and rhizosphere soils. In maize stalk residues, metabarcoding of 24 samples from eight fields generated more than 1.0 million 16S ribosomal RNA (16S rRNA) sequences, 2334 bacterial OTUs, and 48 *Fusarium* OTUs; the dominant *Fusarium* species were *F. graminearum*, *F. avenaceum,* and *F. poae*, accounting for 41.3%, 35.4%, and 7.1% of *Fusarium* sequences, respectively^52^. Co‐occurrence networks further revealed negative associations between *Fusarium* taxa and potential antagonists such as *Sphingomonas*, *Sarocladium strictum*, and *Epicoccum nigrum*, suggesting that residue microbiomes can be used to evaluate inoculum reservoirs and identify suppressive microbial indicators. In maize kernels, metagenomic analysis showed that *F. verticillioides* abundance was positively correlated with *Fusarium* ear rot severity, with correlation coefficients of 0.41–0.49, whereas potential protective taxa such as *Talaromyces stipitatus*, *Ustilago maydis*, and *Burkholderia cenocepacia* showed negative associations with disease or pathogen abundance^53^. Rhizosphere studies also support microbiome‐based warning. In continuous maize systems, disease incidence increased with cropping duration, reaching 8.23% for seedling blight, 9.58% for root rot, 13.55% for stem rot, and 12.92% for ear rot, accompanied by shifts in fungal OTU richness and enrichment of potential pathogens such as *Fusarium*, *Gibberella*, *Cladosporium*, *Acremonium*, and *Bipolaris* ^2^. In northern corn leaf blight‐affected maize, healthy and diseased rhizospheres showed significant beta‐diversity differences, and functional metagenomics identified 28 major SEED functional categories, with healthy rhizospheres generally retaining more dominant functions than diseased ones^54^. Therefore, monitoring shifts in beneficial taxa, changes in microbial diversity, increase in pathogen‐relative abundance, or disruption of protective co‐occurrence networks could provide early signals of disease‐conducive conditions.

In black soil maize fields, practical early warning should not depend on a single universal biomarker, because pathogen pressure, microbial community structure, soil properties, climate, and management history jointly regulate maize disease development. A realistic near‐term platform for Northeast China should therefore use a tiered monitoring strategy. Routine field screening could rely on inexpensive soil health indicators and pathogen‐specific quantitative polymerase chain reaction (qPCR) or loop‐mediated isothermal amplification (LAMP) assays, while representative monitoring sites could be subjected to 16S rRNA and internal transcribed spacer (ITS) sequencing to capture broader bacterial and fungal community shifts^50^, ^52^. These microbiome data should be further integrated with soil physicochemical parameters, weather records, cropping history, straw‐return and tillage practices, fertilization regimes, and remote‐sensing signals of crop stress. By combining these multi‐source datasets, artificial intelligence (AI)‐based models could generate regional disease‐risk maps and provide pre‐symptomatic warnings, thereby supporting more precise disease management in maize production systems of the black soil region of Northeast China.

## CONCLUSION AND FUTURE PERSPECTIVES

Maize disease control in the black soil region of Northeast China should be understood as an integrated soil–microbe–plant process rather than a simple pathogen‐eradication problem. Continuous maize cultivation, residue retention, soil organic matter decline, nutrient imbalance, and climate variability jointly reshape pathogen reservoirs, rhizosphere community assembly, and host susceptibility. Therefore, future management should move from reactive chemical control toward preventive ecological regulation that combines black soil conservation, pathogen monitoring, microbiome intervention, and plant immune enhancement. A practical deployment pathway should begin with regional zoning and risk diagnosis. Fields with long‐term maize monoculture, poor aggregate stability, low organic matter, high residue‐borne *Fusarium* inoculum, or a history of stalk, root, or ear rot should be prioritized for integrated regulation. Before sowing, soil‐health indicators, pathogen assays, cropping history, and weather‐risk information can guide cultivar selection, seed treatment, residue management, and the choice of microbial products. At sowing and early growth stages, *Bacillus*, *Trichoderma*, or locally adapted SynComs can be delivered through seed coatings, soil amendments, or carrier‐based formulations to establish early rhizosphere protection, while MIGS‐ and phage‐based strategies may be considered when bacterial and fungal pathogens are diagnosed.

Future research should strengthen mechanistic and field‐scale validation. Multi‐year trials across representative black soil regions are needed to compare disease incidence, yield stability, microbial persistence, soil‐health recovery, cost‐effectiveness, and biosafety. Mechanistic studies should link maize genotype, root exudates, microbial selection, beneficial microbe recruitment, pathogen inhibition, and plant immune responses. Particular attention should be paid to identify locally adapted keystone taxa, constructing stable SynComs, improving phage and microbial‐inoculant formulations, and assessing the ecological effects of engineered RNAi‐delivering microbes. Microbiome‐based early warning will be essential for precision deployment. Future platforms should integrate pathogen surveillance, 16S/ITS sequencing, metagenomics or metabolomics, soil physicochemical data, remote sensing, crop‐growth records, and AI‐assisted prediction to detect disease‐conducive shifts before visible symptoms occur. Such decision‐support systems can help match biological control, reduced chemical inputs, rotation, straw management, and organic amendments to local risk conditions. Ultimately, the long‐term goal is to build a resilient crop production system in which soil health restoration, microbiome stability, and inducible plant immunity jointly support sustainable disease management in Northeast China's black soil region.
